# Machine learning algorithms for outcome prediction in (chemo)radiotherapy: An empirical comparison of classifiers

**DOI:** 10.1002/mp.12967

**Published:** 2018-06-13

**Authors:** Timo M. Deist, Frank J. W. M. Dankers, Gilmer Valdes, Robin Wijsman, I‐Chow Hsu, Cary Oberije, Tim Lustberg, Johan van Soest, Frank Hoebers, Arthur Jochems, Issam El Naqa, Leonard Wee, Olivier Morin, David R. Raleigh, Wouter Bots, Johannes H. Kaanders, José Belderbos, Margriet Kwint, Timothy Solberg, René Monshouwer, Johan Bussink, Andre Dekker, Philippe Lambin

**Affiliations:** ^1^ The D‐lab: Decision Support for Precision Medicine GROW ‐ School for Oncology and Developmental Biology Maastricht University Medical Centre+ Universiteitssingel 40 6229 ER Maastricht The Netherlands; ^2^ Department of Radiation Oncology GROW, School for Oncology and Developmental Biology Maastricht University Medical Center Maastricht The Netherlands; ^3^ Department of Radiation Oncology Radboud University Medical Center Nijmegen The Netherlands; ^4^ Department of Radiation Oncology University of California San Francisco San Francisco CA USA; ^5^ Department of Radiation Oncology University of Michigan Ann Arbor Michigan USA; ^6^ Department of Radiation Oncology The Netherlands Cancer Institute–Antoni van Leeuwenhoek Hospital Amsterdam The Netherlands; ^7^ Department of Radiation Oncology (MAASTRO) GROW, School for Oncology and Developmental Biology Maastricht University Medical Center Maastricht The Netherlands; ^8^ Institute for Hyperbaric Oxygen (IvHG) Arnhem The Netherlands

**Keywords:** classification, machine learning, outcome prediction, predictive modeling, radiotherapy

## Abstract

**Purpose:**

Machine learning classification algorithms (classifiers) for prediction of treatment response are becoming more popular in radiotherapy literature. General Machine learning literature provides evidence in favor of some classifier families (random forest, support vector machine, gradient boosting) in terms of classification performance. The purpose of this study is to compare such classifiers specifically for (chemo)radiotherapy datasets and to estimate their average discriminative performance for radiation treatment outcome prediction.

**Methods:**

We collected 12 datasets (3496 patients) from prior studies on post‐(chemo)radiotherapy toxicity, survival, or tumor control with clinical, dosimetric, or blood biomarker features from multiple institutions and for different tumor sites, that is, (non‐)small‐cell lung cancer, head and neck cancer, and meningioma. Six common classification algorithms with built‐in feature selection (decision tree, random forest, neural network, support vector machine, elastic net logistic regression, LogitBoost) were applied on each dataset using the popular open‐source *R* package *caret*. The *R* code and documentation for the analysis are available online (https://github.com/timodeist/classifier_selection_code). All classifiers were run on each dataset in a 100‐repeated nested fivefold cross‐validation with hyperparameter tuning. Performance metrics (AUC, calibration slope and intercept, accuracy, Cohen's kappa, and Brier score) were computed. We ranked classifiers by AUC to determine which classifier is likely to also perform well in future studies. We simulated the benefit for potential investigators to select a certain classifier for a new dataset based on our study (*pre‐selection* based on other datasets) or estimating the best classifier for a dataset (*set‐specific selection* based on information from the new dataset) compared with uninformed classifier selection (random selection).

**Results:**

Random forest (best in 6/12 datasets) and elastic net logistic regression (best in 4/12 datasets) showed the overall best discrimination, but there was no single best classifier across datasets. Both classifiers had a median AUC *rank* of 2. Preselection and set‐specific selection yielded a significant average AUC improvement of 0.02 and 0.02 over random selection with an average AUC *rank* improvement of 0.42 and 0.66, respectively.

**Conclusion:**

Random forest and elastic net logistic regression yield higher discriminative performance in (chemo)radiotherapy outcome and toxicity prediction than other studied classifiers. Thus, one of these two classifiers should be the first choice for investigators when building classification models or to benchmark one's own modeling results against. Our results also show that an informed preselection of classifiers based on existing datasets can improve discrimination over random selection.

## 1 Introduction

Machine learning algorithms for predicting (chemo)radiotherapy outcomes (e.g., survival, treatment failure, toxicity) are receiving much attention in literature, for example, in decision support systems for precision medicine.^1^, ^2^ Currently, there is no consensus on an optimal classification algorithm. Investigators select algorithms for various reasons: the investigator's experience, usage in literature, data characteristics and quality, hypothesized feature dependencies, availability of simple implementations, and model interpretability. One objective criterion for selecting a classifier is to maximize a chosen performance metric, for example, discrimination (expressed by the area under the receiver operating characteristic curve, AUC). Here, we discuss the performance of binary classifiers in (chemo)radiotherapy outcome prediction, that is, algorithms that predict whether or not a patient has a certain outcome. We empirically study the behavior of existing simple implementations of classifiers on a range of (chemo)radiotherapy outcome datasets to possibly identify a classifier with overall maximal discriminative performance. This is a relevant question for investigators who search for a rational basis to support their choice of a classifier or who would like to compare their own modeling results to established algorithms.

We employ various open‐source *R* packages interfaced with the *R* package *caret* 3 (version 6.0‐73) that is readily available for investigators and has shown to produce competitive results.^4^ With our results, we also wish to provide guidance in the current trend to delegate modeling decisions to Machine learning algorithms.

Large‐scale studies in the general Machine learning literature^4^, ^5^, ^6^ provide evidence in favor of some classifier families [random forest (*rf*), support vector machine (*svm*), gradient boosting machine (*gbm*)] in terms of classification performance. In our study, we investigate how these results translate to (chemo)radiotherapy datasets for treatment outcome prediction/prognosis. To the best of our knowledge, this is the first study to investigate classifier performance on a wide range of such datasets. The studied features are clinical, dosimetric, and blood biomarkers.

Within the framework of existing classifier implementations, we attempt to answer three research questions:

1. Is there a superior classifier for predictive modeling in (chemo)radiotherapy?
2. How dataset dependent is the choice of a classifier?
3. Is there a benefit of choosing a classifier based on empirical evidence from similar datasets (*preselection*)?

Parmar et al.^7^ compared multiple classifiers and feature selection methods (i.e., *filter*‐based feature selection) on *radiomics* data using the *caret* package. We build upon this work and extend the analysis to 12 datasets outside the *radiomics* domain. We omit *filter* methods because all classifiers in our study comprise built‐in feature selection methods (i.e., *embedded* feature selection) and the main advantage of *filter* methods, i.e. low computational cost per feature, is not relevant for our datasets with only modest numbers of features.

## 2 Materials and methods

### 2.A. Data collection

Twelve datasets (3496 patients) with treatment outcomes described in previous studies were collected from public repositories (www.cancerdata.org) or provided by collaborators. Table 1 characterizes these datasets. Given availability, some datasets consist of subsamples of or contain fewer/more patients and/or features than the cohorts described in the original studies. Two datasets were excluded after a preliminary analysis (these datasets are also not mentioned in Table 1) where none of the studied classifiers resulted in an average AUC above 0.51, which is evidence that they contain no discriminative power. Datasets without discriminative power are not suitable for this analysis as we would be unable to determine differences in discriminative performance across classifiers. The patient cohorts of 2 datasets, Wijsman et al.^20^, ^21^, partially overlap but each dataset lists a different outcome (esophagitis and pneumonitis). Datasets were anonymized in the analysis because their identity is not relevant for interpreting the results and to encourage investigators to share their datasets.

**Table 1:** Dataset characteristics. The number of features is determined before preprocessing

Dataset	Disease	Outcome	Prevalence (in %)	Patients	Features	Feature types	Source
Belderbos et al. (2005)^8^	Non‐small‐cell lung cancer	Grade ≥ 2 acute esophagitis	27	156	22	Clinical, dosimetric, blood	Private
Bots et al. (2017)^9^	Head and neck cancer	2‐yr overall survival	42	137	10	Clinical, dosimetric	Private
Carvalho et al. (2016)^10^	Non‐small‐cell lung cancer	2‐yr overall survival	40	363	18	Clinical, dosimetric, blood	Public^11^
Janssens et al. (2012)^12^	Laryngeal cancer	5‐yr regional control	89	179	48	Clinical, dosimetric, blood	Private
Jochems et al. (2016)^13^	Non‐small‐cell lung cancer	2‐yr overall survival	36	327	9	Clinical, dosimetric	Private
Kwint et al. (2012)^14^	Non‐small‐cell lung cancer	Grade ≥ 2 acute esophagitis	61	139	83	Clinical, dosimetric, blood	Private
Lustberg et al. (2016)^15^, ^16^	Laryngeal cancer	2‐yr overall survival	83	922	7	Clinical, dosimetric, blood	Private
Morin et al. (forthcoming)	Meningioma	Local failure	36	257	18	Clinical	Private
Oberije et al. (2015)^17^	Non‐small‐cell lung cancer	2‐yr overall survival	17	548	20	Clinical, dosimetric	Public^18^
Olling et al. (2017)^19^	Small and non‐small‐cell lung cancer	Odynophagia prescription medication	67	131	47	Clinical, dosimetric	Private
Wijsman et al. (2015)^20^	Non‐small‐cell lung cancer	Grade ≥ 2 acute esophagitis	36	149	11	Clinical, dosimetric, blood	Private
Wijsman et al. (2017)^21^	Non‐small‐cell lung cancer	Grade ≥ 3 radiation pneumonitis	14	188	18	Clinical, dosimetric, blood	Private

**Figure 1:**
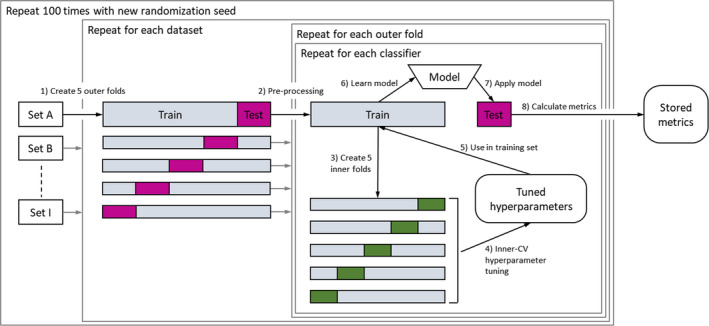
Experimental design: each dataset is split into five stratified outer folds (step 1). For each of the folds, the data are preprocessed (imputation, dummy coding, deleting zero variance features, rescaling) (step 2). The hyperparameters are tuned in the training set via a fivefold inner CV (steps 3–5). Based on the selected hyperparameters, a model is learned on the training set (step 6) and applied on the test set (step 7). Performance metrics are calculated on the test set (step 8) and stored for all outer folds. This process is repeated 100 times for each classifier. Randomization seeds are stable across classifiers within a repetition to allow pairwise comparison. [Color figure can be viewed at wileyonlinelibrary.com]

Nonbinary outcomes were dichotomized, for example, overall survival was translated into 2‐yr overall survival in the dataset of Carvalho et al.^10^. Missing data were imputed for training and test sets (the splitting of datasets into training and test sets is described in Section 2.C) by medians for continuous features and modes for categorical features based on the training set. Basing the imputation on the training set avoids information leakage from test to training sets. Categorical features in training and test sets were dummy coded, that is, representing categorical features as a combination of binary features, based on the combined set for classifiers that cannot handle categorical features (Table 2). Dummy coding on the combined set ensures that the coding represents all values observed in a dataset. Features with zero variance in training sets were deleted in the training set and in the corresponding test set. In addition, we removed near‐zero variance features for *glmnet* to avoid the classifier implementation from crashing during the fitting process. Features in training sets were rescaled to the interval [0,1] and the same transformation was applied to the corresponding test sets. Rescaling is needed for certain classifiers, e.g., *svmRadial*. All these operations (imputation, dummy coding, deleting (near‐)zero variance features, rescaling) were performed independently for each pair of training and test sets (step 2 in Fig. 1).

**Table 2:** Classifier characteristics

Classifier	*caret* 3 label	*R* package	Requires dummy coding	Tuned hyperparameters
Elastic net logistic regression	*glmnet*	*glmnet* 24	Yes	*α*,* λ*
Random forest	*rf*	*randomForest* 25	No	*mtry*
Single‐hidden‐layer neural network	*nnet*	*nnet* 26	No	*size*,* decay*
Support vector machine with radial basis function (RBF) kernel	*svmRadial*	*kernlab* 27	Yes	*σ*,* C*
LogitBoost	*LogitBoost*	*caTools* 28	Yes	*nlter*
Decision tree	*rpart*	*rpart* 29	No	*cp*

### 2.B. Classifiers

Six common classifiers were selected and their implementations were used via their interfacing with the open‐source *R* package *caret*. The selection includes classifiers frequently used in medical data analysis and advanced classifiers such as random forests or neural networks.

□ Elastic net logistic regression is a regularized form of logistic regression, which models additive linear effects. The added shrinkage regularization (i.e., feature selection) makes it is suitable for datasets with many features while maintaining the interpretability of a standard logistic regression.
□ Random forests generate a large number of decision trees based on random subsamples of the training set while also randomly varying the features used in the trees. Random forests allow modeling nonlinear effects. A random forest model is an ensemble of many decision tree models and is, therefore, difficult to interpret.
□ Single‐hidden‐layer neural networks are simple versions of multilayer perceptron neural network models, which are currently popularized by deep neural network applications in machine learning. In the hidden layer, auxiliary features are generated from the input features which are then used for classification. The weights used to generate auxiliary features are derived from the training set. The high number of weights requires more training data than other simpler algorithms and reduces interpretability. However, if sufficient data are available, complex relationships between features can be modeled.
□ Support vector machines with a radial basis function (RBF) kernel transform the original feature space to attain a better separation between classes. This transformation, however, is less intuitive than linear SVMs where a separating hyperplane is in the original feature space.
□ LogitBoost (if used with decision stumps as in this paper) learns a linear combination of multiple single feature classifiers. Training samples that are misclassified in early iterations of the algorithm are given a higher weight when determining further classifiers. The final model is a weighted sum of single feature classifiers. Similar to random forests, it builds an ensemble of models which is difficult to interpret.
□ A decision tree iteratively subdivides the training set by selecting feature cutoffs. Decision trees can model nonlinear effects and are easily interpretable as long as the tree depth is low.

Classifier details can be found in general Machine learning textbooks.^22^, ^23^ Table 2 further characterizes these classifiers. We use the option in *caret* to return class probabilities for all classifiers, including nonprobabilistic classifiers like *svmRadial*. Classifier hyperparameters, that is, model‐intrinsic parameters that need to be adjusted to the studied data prior to modeling, were tuned for each classifier using a random search: 25 randomly chosen points in the hyperparameter space are evaluated and the point with the best performance metric (we chose the AUC in this study) is selected. The boundaries of the hyperparameter space are given in *caret*.

### 2.C. Experimental design

For each classifier, test set (or *out‐of‐sample*) performance metrics (AUC, Brier score, accuracy, and Cohen's kappa) were estimated for each of the 12 datasets. The performance metric estimator was the average performance metric computed from the outer test folds in a nested and stratified fivefold cross‐validation (CV). The experiment was repeated 100 times. The 100 times repeated nested cross‐validation yields a better estimate of the true test set performance by randomly simulating many scenarios with varying training and test set compositions.

The experimental design is depicted in Fig. 1: Each dataset was split into five random subsamples stratified for outcome classes (step 1 in Fig. 1), each of them acting once as a test set and four times as a part of a training set. The number of inner and outer folds was set to 5 following standard practice^23^(p 242). Data preprocessing is done per pair of training and test sets (step 2; see details in section *Datasets*). The models were trained on the training set (step 6) and applied on the test set (step 7) to compute the performance metrics for the test set (step 8) resulting in five estimates per performance metric (i.e., 1 per outer fold). During the training in each outer fold, the best tuning parameters were selected from the random search (see section *Classifiers*) according to the maximum AUC of an inner fivefold CV. In the inner CV, the training set was again split into five subsamples and models with different tuning parameters were compared (steps 3–5). The nested fivefold CV was repeated 100 times with different randomization seeds which are used, for example, for generating the outer folds in step 1. Note that the performance metrics computed on the outer test folds of any two classifiers can be analyzed by pairwise comparison because the classifiers were trained (step 6) and tested (step 7) on the same training and test sets for a specific dataset within each of the 100 repetitions.

The mean AUC, Brier score, accuracy, and Cohen's kappa were computed from the five estimates of the fivefolds in the outer CV. Calibration intercept and slope were computed from a linear regression of outcomes and predicted outcome probabilities for each of the five outer folds. To attain aggregated calibration metrics over the five outer folds of the CV, the mean absolute differences from 0 and 1 were computed for the calibration intercept and slope, respectively. Classifier rankings were computed per dataset and repetition by ordering the classifiers’ CV‐mean AUC (i.e., the average AUC for five test sets) in descending order and then assigning the ranks from 1 to 6. Using CV‐mean AUCs and CV‐mean AUC *ranks*, we answer research questions 1 and 2. We chose AUC for the analysis following Steyerberg et al.^30^ They emphasize the importance of discrimination and calibration metrics when assessing prediction models. For the simplicity, we restricted the extended analysis to discrimination (AUC) but also report results for calibration and other metrics in appendix A.

To address the question of preselection (research question 3), we assess the advantage of choosing a classifier based on performance metrics from similar datasets, which we call *preselection* below. To estimate the benefit of our classifier preselection for a new dataset and to compare it to alternative strategies, the results of the experiment above were used as input for a simulation. For each outer fold of the 1200 fivefold CVs (12 datasets × 100 repetitions × 5‐folds = 6000‐folds), three classifier selections were made and tested on the test set that belongs to the specific outer fold:

□ preselecting the classifier according to the average AUC *rank* in all other datasets (excluding all folds from the current dataset),
□ selecting the classifier that performed best in the inner CV on the training set,
□ randomly selecting a classifier.

Preselecting the classifier for one dataset that had the best average AUC *rank* in the other datasets simulates the scenario in which an investigator bases their classifier choice on empirical evidence as is reported in this manuscript. Randomly selecting a classifier represents the case where an investigator chooses a classifier without any prior knowledge about the dataset that (s)he is about to analyze. Selecting the tuned classifier with best inner CV performance corresponds to evaluating multiple classifiers on the training dataset and thus including dataset‐specific information in the classifier selection. The performance metrics are averaged over all 500 outer folds (5‐folds × 100 repetitions) for each of the 12 datasets.

The documented *R* code used for the analysis is available online.^31^

## 3 Results

Running 1 nested fivefold cross‐validation and computing the metrics on one dataset for all six classifiers allows one comparison of classifiers. This was applied on 12 different datasets, with each run repeated 100 times for a total of 1200 comparisons. The total computation time was approximately 6 days on an Intel Core i5‐6200U CPU (or 15 s per classifier per dataset per outer fold, on average).

The results are presented and discussed threefold:

1. results aggregated over all datasets and repetitions to determine the presence of a superior classifier,
2. separate results for each dataset but aggregated over repetitions to determine dataset dependency,
3. a simulation of classifier selection methods in new datasets to estimate the relative effect of classifier preselection.

The detailed analysis is restricted to the classifiers’ discriminative performance according to the AUC. Results for the remaining metrics (Brier score, calibration intercept/slope, accuracy, and Cohen's kappa) are reported in Appendix A.

### 3.A. Results aggregated over all datasets

**Figure 2:**
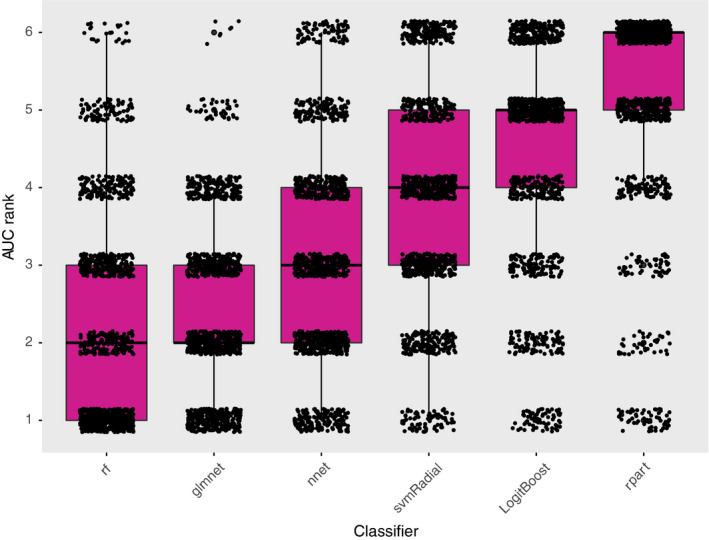
Box and scatterplot of the AUC *rank* (lower being better) per outer fivefold CV aggregated over all datasets and repetitions (12 datasets × 100 repetitions = 1200 data points per classifier). [Color figure can be viewed at wileyonlinelibrary.com]

**Figure 3:**
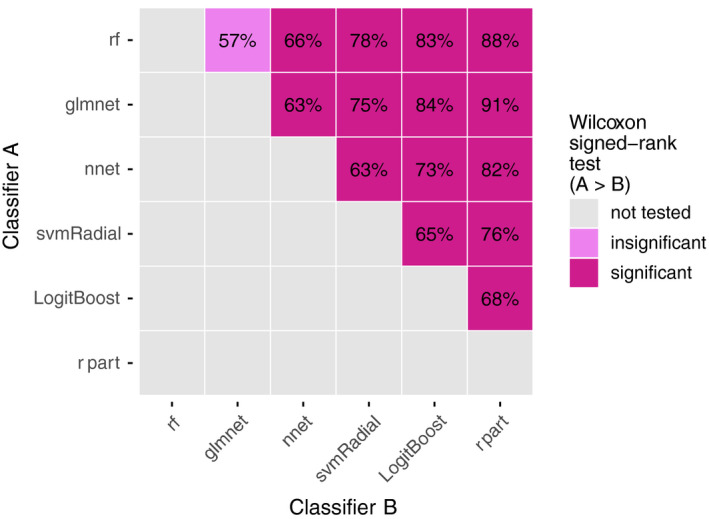
Pairwise comparisons of each classifier pair (12 datasets × 100 repetitions = 1200 comparisons per pair). The numbers in the plot indicate how often classifier A (*y*‐axis) achieved an AUC greater than classifier B (*x*‐axis). The color indicates whether the increased AUCs by classifier A are statistically significant (violet), insignificant (light violet), or have not been tested (gray). The significance cutoff was set to the 0.05‐level (one‐sided Wilcoxon signed‐rank test, Holm–Bonferroni correction for 15 tests). [Color figure can be viewed at wileyonlinelibrary.com]

Figure 2 shows the distribution of classifier rankings based on the average AUC (12 datasets × 100 repetitions = 1200 data points per classifier). Figure 3 depicts pairwise comparisons for each classifier pair (1200 comparisons per pair). The numbers in the plot indicate how often classifier A (*y*‐axis) achieved an AUC greater than classifier B (*x*‐axis). Coloring indicates whether the increased AUCs of classifier A are statistically significant (violet) or not (light violet). Untested pairs are colored gray. The significance cutoff was set to the 0.05 level (one‐sided Wilcoxon signed‐rank test, Holm–Bonferroni correction for 15 tests).

*rf* and *glmnet* showed the best median AUC *rank*, followed by *nnet*,* svmRadial*,* LogitBoost*, and *rpart* (Fig. 2). At the low end of the ranking, *rpart* showed poor discriminative performance. Manual inspection of the *rpart* models showed that *rpart* frequently returns empty decision trees for particular sets (for 34%, 19%, 68%, 35%, 58% of all outer folds for *sets D*,* E*,* G*,* K*,* L*, respectively). In pairwise comparisons, *rf* and *glmnet* significantly outperformed all other classifiers (Fig. 3). *rf* exhibited a small but statistically insignificant better AUC *rank* than *glmnet*.

The results in Figs. 2 and 3 indicate the existence of a significant classifier ranking for these datasets. However, the considerable spread per classifier in Fig. 2 and the low pairwise comparison percentages (between 57% and 91% in Fig. 3) also suggest a yet unobserved dependency for classifier performance. To this end, the relationship between datasets and varying classifier performance is investigated.

### 3.B. Results separate for each dataset

**Figure 4:**
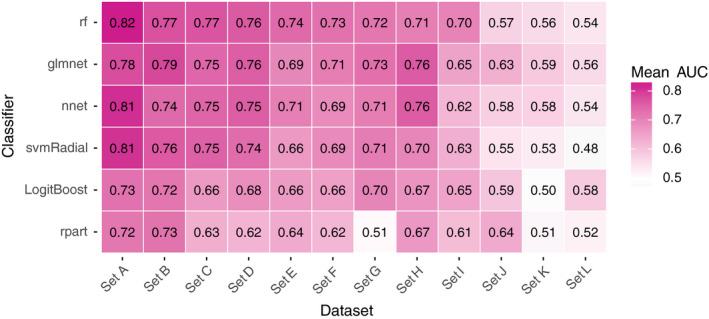
The mean AUC for each pair of classifier and dataset (100 repetitions = 100 data points per pair). [Color figure can be viewed at wileyonlinelibrary.com]

**Figure 5:**
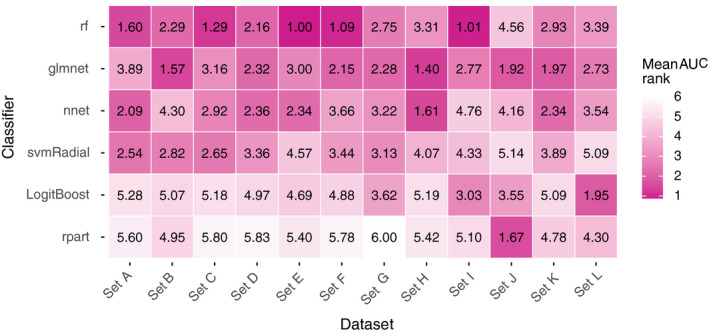
The mean *rank* derived from the AUC (100 repetitions = 100 data points per pair). [Color figure can be viewed at wileyonlinelibrary.com]

Figure 4 shows the average AUC for each pair of classifier and dataset (100 repetitions = 100 data points per pair). Figure 5 depicts the average *rank* derived from the AUC (100 data points per pair).

*rf* and *glmnet* generally yielded higher AUC values and AUC *ranks* per dataset (Figs. 4 and 5). However, this observation is not consistent over all datasets: e.g., *nnet* outperforms *rf* in *sets H*,* J*, and *K,* and *svmRadial* outperformed *glmnet* in *sets A* and *C*.

The results in the Figs. 4 and 5 indicate that dataset‐specific properties impact the discriminative performance of classifiers. These results challenge our proposition that one can preselect classifiers for predictive modeling in (chemo)radiotherapy based on representative datasets from the same field.

### 3.C. Effects of empirical classifier preselection on discriminative performance

Table 3 lists, for each dataset, the name and average AUCs, that is, averaged over all 100 repetitions, for random classifier selection, classifier preselection, and set‐specific classifier selection.

**Table 3:** For each dataset, the AUC *rank* averaged over all repetitions when (a) randomly selecting a classifier (Random classifier), (b) preselecting the classifier with the average best AUC *rank* in all other datasets, that is, without any information about the current dataset (Preselected classifier), (c) selecting the classifier that yielded the highest AUC in the inner CV (Set‐specific classifier). Improvements in average AUC and average AUC *rank* compared to (a) are reported. The average AUC improvements by preselection and set‐specific selection were tested for statistical significance (*P* < 0.05, one‐sided Wilcoxon signed‐rank test) and found to be statistically significant (*). No other statistical tests besides the two aforementioned tests were conducted

Dataset	Random classifier	Preselected classifier				Set‐specific classifier		
	Rank	Name	Rank		AUC	Rank		AUC
	Mean		Mean	Increase	Increase	Mean	Increase	Increase
Set A	3.59	*glmnet*	3.64	−0.05	0.00	3.10	0.49	0.02
Set B	3.48	*rf*	2.92	0.56	0.02	3.31	0.17	0.01
Set C	3.50	*glmnet*	3.12	0.37	0.03	2.78	0.72	0.03
Set D	3.57	*rf*	2.60	0.97	0.04	3.31	0.26	0.02
Set E	3.53	*glmnet*	3.35	0.18	0.01	1.75	1.78	0.05
Set F	3.39	*rf*	1.89	1.50	0.04	2.58	0.81	0.03
Set G	3.47	*rf*	2.99	0.47	0.04	3.52	−0.06	0.01
Set H	3.44	rf	3.81	−0.37	0.00	1.70	1.74	0.05
Set I	3.45	rf	1.59	1.86	0.06	1.72	1.73	0.05
Set J	3.52	*rf*	4.18	−0.66	−0.02	3.41	0.11	0.00
Set K	3.50	*rf*	3.33	0.16	0.01	3.20	0.30	0.01
Set L	3.58	*rf*	3.50	0.08	0.01	3.66	−0.08	0.00
**Mean**	**3.50**		**3.08**	**0.42**	**0.02** ^*^	**2.84**	**0.66**	**0.02** ^*^

The preselection procedure always results in *rf* or *glmnet*. The mean benefit of empirically preselecting a classifier is small: the AUC improvement ranges between −0.02 and 0.06 with a mean of 0.02. In a pairwise comparison over all datasets (*P* < 0.05, one‐sided Wilcoxon signed‐rank test), the AUC values by preselection were significantly larger than the AUC values by random selection. The AUC *rank* improves by 0.42 on average. Including dataset‐specific information by inner CV yields a mean AUC improvement of 0.02 and improves the *rank*, on average, by 0.66. In a pairwise comparison of set‐specific and random classifier selection over all datasets (*P* < 0.05, one‐sided Wilcoxon signed‐rank test), the AUC increase was also statistically significant.

Given this simulation, the expected benefit of preselecting a classifier for a new dataset based on results from (chemo)radiotherapy‐specific numerical studies is limited with an average increase in AUC of 0.02.

## 4 Discussion

Our results suggest that there is indeed an overall ranking of classifiers in (chemo)radiotherapy datasets, with *rf* and *glmnet* leading the ranking. However, we also observe that the performance of a classifier depends on the specific dataset. Preselecting classifiers based on evidence from related datasets would, on average, provides a benefit for investigators because it increases discriminative performance. An increase in average discriminative performance is desirable in that an investigator would be less likely to discard their data because of a perceived absence of predictive or prognostic value. The estimated 0.02 mean AUC improvement might appear small, but it comes “for free” with classifier selection based on empirical evidence from multiple radiotherapy datasets. Furthermore, the 0.02 AUC improvement is relative to random classifier selection. If an investigator had initially chosen *rpart,* which is the overall worst performing classifier in our study, switching to the preselected classifier would result in an average AUC increase of 0.07. Switching from LogitBoost, which is the second worst performing classifier in our study, to the preselected classifier would result in an average AUC increase of 0.04.

The results in Table 3 show that classifier preselection and set‐specific classifier selection, on average, yield the same AUC increase. We think that the usefulness of set‐specific classifier selection is dependent on the size of the training set: classifier preselection is preferable for small datasets, set‐specific classifier selection is better for larger datasets. Classifier preselection represents choosing classifiers using evidence from a large collection of similar datasets from the general radiotherapy outcome domain. Set‐specific classifier selection represents choosing classifiers based on the training set, which is a considerably smaller evidence base but comes from the patient group under investigation. If the training dataset is too small, selecting classifiers based on results from other datasets might be less‐error prone. On the contrary, if an investigator has collected a large dataset, they have the option to conduct set‐specific classifier selection (with all six classifiers) for their training data using our documented *R* code.^31^

In Table 3, one can observe that the preselected classifier is mostly *rf* and sometimes *glmnet*. To understand this behavior, consider dataset *A*:* glmnet* was preselected for *set A* by selecting the classifier with the best average AUC *rank* in all other sets (excluding *set A*). Note that, for all 12 datasets together, the average AUC *rank* for *rf* is only slightly better than for *glmnet* (2.28 for *rf* and 2.43 for *glmnet*; the average of the rows in Fig. 5). Since *glmnet* performs badly while *rf* performs best in *set A*, excluding this information leads to a better average AUC *rank* for *glmnet* and a worse average AUC *rank* for *rf* in the remaining 11 datasets. As a consequence, *glmnet* becomes the preselected classifier for this dataset. A similar behavior is observed for *sets C* and *E* but not in *sets D*,* F*,* I*, where *glmnet* also performs worse than *rf* but the difference between both classifiers is smaller and does not induce a switch in the preselected classifier.

The result that classifier preselection is as good as set‐specific selection in the studied datasets does *not* imply that one *cannot* determine a better classifier for a new dataset. Our implementation of set‐specific classifier selection only evaluates the performance of various classifiers but does not directly take into account properties of the dataset itself. For example, if an investigator collected a dataset in which the outcome has a quadratic dependency on a feature, *glmnet* would not be able to capture this relation (since it models only linear effects) but *rf* would. However, preselecting a classifier based on results from other (chemo)radiotherapy datasets works well on average. Furthermore, including set‐specific classifier selection complicates the modeling process and, therefore, might not be desirable.

In this study, we collected 12 datasets for different treatment sites, that is, (non‐)small‐cell lung cancer, head and neck cancer, meningioma with different outcomes, that is, survival, pneumonitis, esophagitis, odynophagia, and regional control. However, this collection is certainly not a complete representation of treatment outcome datasets analyzed in the field of radiotherapy. Furthermore, we only studied one implementation of classifiers, while classifier performance may vary between implementations. Past studies, however, indicate that classifier implementations in *R* interfaced with *caret* are competitive.^4^ Given the apparent lack of comparative classifier studies in radiotherapy, our intention has been to provide numerical evidence for classifier selection to investigators even though our analysis is not exhaustive.

We intentionally limited the analysis to classifier selection while ignoring factors such as the investigator's experience, usage in literature, hypothetical feature dependencies, and model interpretability. This restriction imitates the current trend to delegate modeling decisions to Machine learning algorithms and/or nondomain experts. Nonetheless, we feel the need to emphasize that including these factors has merit. Furthermore, expertise on a specific classifier could warrant its selection: Lavesson and Davidsson^32^ observed in a study on eight datasets from different research domains that the impact of hyperparameter tuning exceeds that of classifier selection. Therefore, the investigator could tune a classifier for better performance by also tuning the hyperparameters outside the subset of hyperparameters tuneable inside *caret*. Even in those cases, however, we suggest comparing these results to simpler implementations of *rf* and *glmnet* as these classifiers on average have the best discriminative performance according to this study.

Finally, for the clinical implementation of classifiers, model interpretability is arguably a major requirement^33^: this view is also convincingly motivated by Caruana et al.^34^ Fortunately, our study shows that *glmnet*, which is an intuitive classifier, is also one of the best performing classifiers.

## 5 Conclusion

We have modeled treatment outcomes in 12 datasets using six different classifier implementations in the popular open‐source software *R* interfaced with the package *caret*. Our results provide evidence that the easily interpretable elastic net logistic regression and the complex random forest classifiers generally yield higher discriminative performance in (chemo)radiotherapy outcome and toxicity prediction than the other classifiers. Thus, one of these two classifiers should be the first choice for investigators to build classification models or to compare one's own modeling results. Our results also show that an informed preselection of classifiers based on existing datasets improves discrimination over random selection.

## Conflicts of Interest

Andre Dekker, Johan van Soest, and Tim Lustberg are founders and shareholders of Medical Data Works B.V., which provides consulting on medical data collection and analysis projects. Cary Oberije is CEO of ptTheragnostic B.V. Philippe Lambin is member of the advisory board of ptTheragnostic B.V.

## Appendix A

### A.1

Table AI lists performance metrics per classifier. These values are averaged over all repetitions and datasets (100 repetitions × 12 datasets = 1200 data points each). Accuracy and Cohen's kappa were computed at the 0.5 cutoff. Calibration fails in some outer folds for every classifier resulting in either large or undefined values for intercept and/or slope. This failure occurs frequently with *nnet* and *rpart*. Undefined (NaN) values are excluded when calculating the median.

**Table AI:** Median performance metrics per classifier aggregated over repetitions and datasets (1200 data points each). Undefined (NaN) values are excluded when calculating the median

Classifier	AUC	Brier score	Accuracy	Cohen's kappa	Calibration intercept error	Calibration slope error
*rf*	0.72	0.17	0.72	0.10	0.12	0.37
*glmnet*	0.72	0.18	0.72	0.14	0.26	0.68
*nnet*	0.71	0.21	0.69	0.11	0.36	0.96
*svmRadial*	0.69	0.18	0.72	0.06	0.26	0.86
*LogitBoost*	0.66	0.23	0.68	0.18	0.22	0.60
*rpart*	0.63	0.20	0.71	0.16	0.21	0.56
